# Expression and function of PDGF-C in development and stem cells

**DOI:** 10.1098/rsob.210268

**Published:** 2021-12-01

**Authors:** Yi Tian, Ying Zhan, Qin Jiang, Weisi Lu, Xuri Li

**Affiliations:** ^1^ State Key Laboratory of Ophthalmology, Zhongshan Ophthalmic Center, Sun Yat-sen University, Guangdong Provincial Key Laboratory of Ophthalmology and Visual Science, Guangzhou 510060, People’s Republic of China; ^2^ Ophthalmic Department, Affiliated Eye Hospital of Nanjing Medical University, Nanjing, People's Republic of China

**Keywords:** platelet-derived growth factor C, development, embryogenesis, organogenesis, stem cell, pluripotency

## Abstract

Platelet-derived growth factor C (PDGF-C) is a relatively new member of the PDGF family, discovered nearly 20 years after the finding of platelet-derived growth factor A (PDGF-A) and platelet-derived growth factor B (PDGF-B). PDGF-C is generally expressed in most organs and cell types. Studies from the past 20 years have demonstrated critical roles of PDGF-C in numerous biological, physiological and pathological processes, such as development, angiogenesis, tumour growth, tissue remodelling, wound healing, atherosclerosis, fibrosis, stem/progenitor cell regulation and metabolism. Understanding PDGF-C expression and activities thus will be of great importance to various research disciplines. In this review, however, we mainly discuss the expression and functions of PDGF-C and its receptors in development and stem cells.

## Graphical abstract

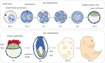

## 1. Introduction

The platelet-derived growth factor (PDGF) family consists of four ligands (PDGF-A, -B, -C and -D) and two receptors (PDGFR-α and PDGFR-β) [1–3]. The PDGFs bind the PDGFRs and trigger their dimerization, which induces phosphorylation of the tyrosine residues in the intracellular domain of the receptors [4]. The phosphorylated receptors activate various downstream pathways, including Ras-MAPK, PI3 K and PLC-γ signalling, and participate in diverse physiological and pathological processes, such as embryonic development, angiogenesis, tumour growth, stem cell regulation and metabolism [2,5–7].

PDGF-C was discovered in 2000 [8], about 20 years after the finding of PDGF-A and PDGF-B [1,3]. PDGF-C mainly binds to PDGFR-α [8]. When PDGFR-β is co-expressed with PDGFR-α, it can be engaged by PDGF-C as well [9]. Studies from the past 20 years or so have demonstrated important roles of PDGF-C in diverse biological processes, such as development, tumour growth, angiogenesis, wound healing, tissue remodelling, fibrosis, atherosclerosis, metabolism and stem/progenitor cell regulation [5,10–16]. In this review, however, we mainly discuss the roles and expressions of PDGF-C and its receptors in human and murine development and stem cells.

## 2. Expression of PDGF-C and its receptors in embryonic development and adults

**Figure 1.**
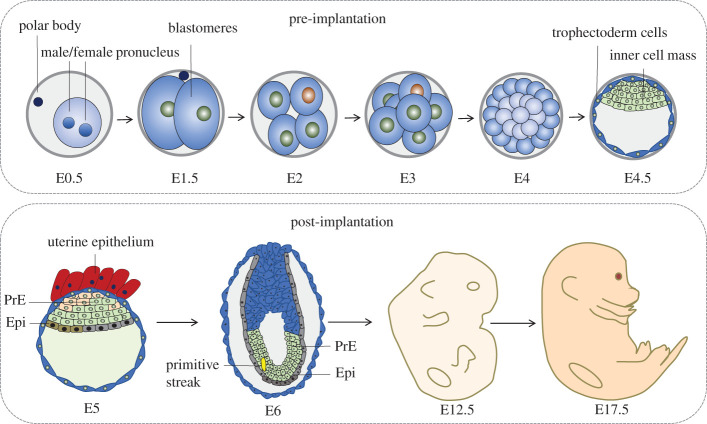
Stages of mouse embryogenesis. Mouse embryogenesis starts at fertilization (E0.5), and gives rise to a zygote (E1.0), which undergoes sequential cleavages to form a blastomere (E1.0–E2.5). The blastomere then undergoes compaction and polarization to form a blastocyst (E2.5–E3.5). The outer trophoblast layer of the blastocyst separates from the inner cell mass (ICM) and the blastocyst expands. The late blastocyst is implanted in the uterine wall (E4.5–E5.0), after which the embryo undergoes gastrulation (E6.0) and forms the ectoderm, mesoderm and endoderm germ layers. Organogenesis starts at late gastrulation and continues until birth (E6.0–E18.5).

Embryonic development (embryogenesis), the process of embryo formation from a zygote and its further growth until birth, entails coordinated spatio-temporal regulation of gene expression, cell division and differentiation [17,18]. In mammals, such as in mice, embryogenesis is divided into pre-implantation and post-implantation stages (figure 1). In pre-implantation stage, the zygote forms a blastocyst, which is subsequently implanted in the uterus, thus entering the post-implantation stage [19] (figure 1). In post-implantation stage, the embryo develops into a gastrula that generates the ectoderm, mesoderm and endoderm germ layers, from which organ development (organogenesis) initiates and continues until birth [20,21] (figure 1).

### 2.1. PDGF-C and its receptors are highly expressed in pre-implantation embryos

In the early stage of human embryonic development, such as in eight-cell stage embryos, *PDGF-C* and its receptors are abundantly expressed [22–24]. In human blastocysts, *PDGF-C* is detected in the inner cell mass (ICM), the pluripotent epiblast (EPI), the extra-embryonic primitive endoderm (PrE) and trophoblast [22] with similar expression levels in PrE and EPI, and a higher level in trophoblast [23]. *PDGFR-α*, the major receptor used by PDGF-C, is detected as early as in four-cell stage embryos and blastocyst. *PDGFR-β*, which can be engaged by PDGF-C when it is co-expressed with *PDGFR-α*, is also detected in four-cell stage embryos [24]. In mice, like in humans, abundant *Pdgf-c* expression is found in early embryonic development. *Pdgf-c* is detected in mouse zygotes and in embryos of two-cell, eight-cell and blastocyst stages as shown by single-cell RNA sequencing [25]. *Pdgf-c* is upregulated in EPI at E4.5 [26,27]. Both PDGFR-α and PDGFR-β are abundantly expressed in mouse zygotes and blastocyst [25,28]. In summary, both PDGF-C and its receptors are abundantly expressed in human and mouse pre-implantation embryos, suggesting the possible effects of PDGF-C on early embryogenesis.

### 2.2. PDGF-C and its receptors are expressed in all the three germ layers in post-implantation embryos

In post-implantation embryos, PDGF-C is highly expressed in all the three germ layers and their derivatives (table 1). In the ectoderm, PDGF-C is expressed in the neural tube and its derivatives, such as in the cerebral cortex, floorplate, spinal cord, cerebellum and hindbrain of the central nervous system (CNS) [29,35,36] (table 1). Expression of PDGF-C is also found in the cephalic neural crest and its derivatives, such as in the eye, follicles, branchial arches and pouches [29,35,36]. In the mesoderm, PDGF-C is expressed in the notochord, somite and their derivatives, such as in the sclerotome, myotome, the mesenchyme surrounding the cavities, bladder, kidney, head and heart [29,35]. In the endoderm, PDGF-C is expressed in the oesophagus, lung, gut and salivary gland [29,35]. PDGF-C is also abundantly expressed at the sites of epidermal opening development leading to the formation of the mouth, nostril and ear [29] (table 1).

**Table 1.** PDGF-C expression in mouse embryos.

	location	time	references
*PDGF-C* ectoderm			
central nervous system	hindbrain, cerebellum, spinal cord, floorplate, ventricular and subventricular zones of cortex, epithelial tissue of choroid plexus, choroid plexus, neuron, glial cell	E9.5	[29,30]
neural crest cell	cartilage, osteoblast, odontoblast, inner ear, head mesenchyme of otic vesicle, frontonasal, medulla of adrenal gland	E9.5	[31]
skin	epidermis, follicle, root sheath, branchial arche, branchial pouche, nasal placode	E9.5	[29]
eye	corneal epithelium, boundary of the eyelid, retinal ganglion cell, retinal pigment epithelial cell	E16.5	[29,30]
mesoderm			
somite	myotome and skeletal muscle, sclerotome, myoblast and muscles of limb bud, nuclei pulposi, epaxial muscles of trunk, myoblast and myocyte of facial and cervical muscles, notochord, myoblast of smooth and skeletal muscles, hypertrophic chondrocyte	E9.5	[29]
kidney	metanephric mesenchyme epithelial branche, nephric tubule and ureteric epithelium, urethra, cortex of the adrenal gland, arterial endothelial and smooth muscle cell	E12	[29,32]
heart	cardiomyocyte, smooth muscle cells of aorta and vena cava	E12.5	[29,33]
testes	coelomic epithelium, gonad-mesonephros boundary	E11.5	[34]
endoderm			
lung	epithelium, the bronchial tubule, smooth muscle, mesenchyme, trachea	E14.5	[29]
gut	stomach, small and large intestine, oesophagus, endodermal mucosal epithelium, mesenchyme, smooth muscle layer	E12.5	[29]
salivary gland	epithelial branches, mesenchyme	E12.5	[29]

Like PDGF-C, PDGFR-α and PDGFR-β are also expressed in the three germ layers and their derivatives (table 2). PDGFR-α is widely expressed in the ectoderm lineage, such as the neural crest [41], neural tube and eye [37,42–44] (table 2). PDGFR-α is also expressed in the mesoderm lineage, including mesenchyme tissues [45], somite and its derivatives [45,46], bladder, kidney [8,47], heart [38,45] and testes [48]. Moreover, PDGFR-α is expressed in the endoderm lineage, such as the lung [49–51] and salivary gland [52,53]. In the ectoderm lineage, PDGFR-β is found in the CNS [53–55] and eye [37]. In the mesoderm lineage, PDGFR-β is detected in the kidney [43], heart [56] and testes [48]. PDGFR-β is also expressed in the endoderm lineage, such as the lung [57–59] (table 2). Thus, the general expression of PDGF-C and its receptors in the three germ layers in post-implantation embryos suggest possible functions of PDGF-C during organogenesis.

**Table 2.** PDGFR-α and PDGFR-β expression in mouse embryos.

	location	references
PDGFR-α		[8,28,37–39]
CNS	forebrain, spinal cord, brain stem, neuron, astrocyte, O-2A cell	
heart	cardiac NCC, epicardium, myocardium	
kidney	mesenchyme, vSMC, mesangium, interstitial cell	
lung	mesenchyme	
eye	choroid, sclera, eyelid, epithelium, retinal astrocyte	
PDGFR-β		[37,40]
CNS	forebrain cortex, choroid plexus, neuron, glial cell, optic nerve, oligodendrocyte progenitor	
heart	cardiac NCC, vSMC, epicardial cell	
kidney	perivascular mesenchyme, vSMCs	
lung	epithelial cell, mesenchymal cell, fibroblast	
eye	retina, Müller cell, glial cell	

### 2.3. Expression of PDGF-C and its receptors in adults

In humans, PDGF-C is generally expressed in most adult organs and tissues, such as in the vasculature, heart, brain, kidney, liver, testes, lung, pancreas, ovary, placenta, skeletal muscle, thymus, prostate gland, adrenal gland, breast, colon, uterus and small intestine [60–64] (table 3). Human PDGFR-α and PDGFR-β are also expressed in most of these organs, such as in the brain, kidney, testes, lung and eye [48,83,84].

**Table 3.** PDGF-C expression in adults.

expression site	references
vascular system	[60,65–69]
EC, vSMC, PC, EPC, vascular fibroblast, MSC, macrophage, platelet, fibroblast	
heart	[33,62]
myocardium, cardiac fibroblast, cardiac myofiber	
neural system	[70–73]
cerebellum neuron, anterior olfactory nucleus, pontine nuclei, neuronal cells of cochlea, astrocyte, microglia, oligodendrocyte, OPC	
kidney	[64,74]
parietal epithelial cells of Bowman's capsule, tubular epithelial cells, Henle's loop, distal tubules, collecting ducts, arterial endothelial cells, interstitial cells	
eye	[75,76]
eyelid, RPE cell, choroid, RGC, retinal inner/outer nuclear layers, ganglion and neuronal cells	
liver	[77–79]
hepatocytes, hepatic stellate cells	
lung	[62,80,81]
proximal airway epithelial cells, SMCs, lung fibroblasts, alveolar macrophages, interstitial cells	
other organs	[8,61–63,82]
pancreas, ovary, placenta, skeletal muscle, thymus, prostate gland, adrenal gland, breast, colon, uterus, small intestine	

In mice, PDGF-C is also widely expressed in various organs and cell types, including the brain, heart, vasculature [29,36], kidney, liver, testes and lung [80] (table 3). In the vascular system, PDGF-C is abundantly expressed in vascular endothelial cells (ECs) [60,65], vascular smooth muscle cells (SMCs) [66] and pericytes (PCs) [85]. PDGF-C is also expressed in mouse monocytes, macrophages, platelets and fibroblasts [67]. In the heart, PDGF-C is detected in mouse cardiac fibroblasts and myofibres [8,33]. In the neural system, PDGF-C is highly expressed in projection neurons, interneurons in the cerebral cortex, choroid plexus, spinal cord neurons [36], cerebellum [70], anterior olfactory nucleus, pontine nuclei [36] and neuronal cells in the cochlea [30]. PDGF-C is also detected in mouse glial cells, such as astrocytes [86], microglia [71] and oligodendrocytes [72]. Other mouse organs expressing PDGF-C include the adrenal gland, colon, duodenum, ovary, placenta, thymus and small intestine [82] (table 3). Mouse PDGFR-α and PDGFR-β are expressed in most organs as well, such as in the heart, brain, lung, kidney, spleen, mammary gland, ovary and testes [87,88] (table 4).

**Table 4.** Expression of PFGFR-α and PDGFR-β in adults.

	references
*PDGFR-α*	[69,87,89,90]
vascular system	
ECs, SMCs, PCs, epicardium, myocardium, endocardium, fibroblasts	
neural system	
cerebral cortex, hippocampus, brainstem, spinal cord, neurons, astrocytes, Schwann cells	
kidney	
mesangial cells, SMCs, glomeruli, tubules	
testes	
Leydig cells	
lung	
ECs, SMCs, alveolar macrophages, airway epithelium	
eye	
RPE cells, retina, corneal epithelium, RGCs	
*PDGFR-β*	[60,91–94]
vascular system	
ECs, SMCs, PCs, myocardium, fibroblasts	
neural system	
hippocampal, cortical neurons, Schwann cells	
kidney	
mesangial cells, parietal epithelial cells, interstitial fibroblasts	
testes	
Leydig cells	
lung	
ECs, SMCs, alveolar epithelium	
eyes	
RPE cells, retinal ECs, corneal fibroblasts, RGCs	

## 3. Regulation of PDGF-C expression

PDGF-C activity must be tightly controlled, and uncontrolled PDGF-C expression has been reported to be associated with numerous pathological conditions, such as choroidal neovascularization [95], chronic myocarditis [96], glomerulosclerosis [64], tissue fibrosis [12,15,97–99], atherosclerosis [100] and various tumours [11,98,101–106].

**Figure 2.**
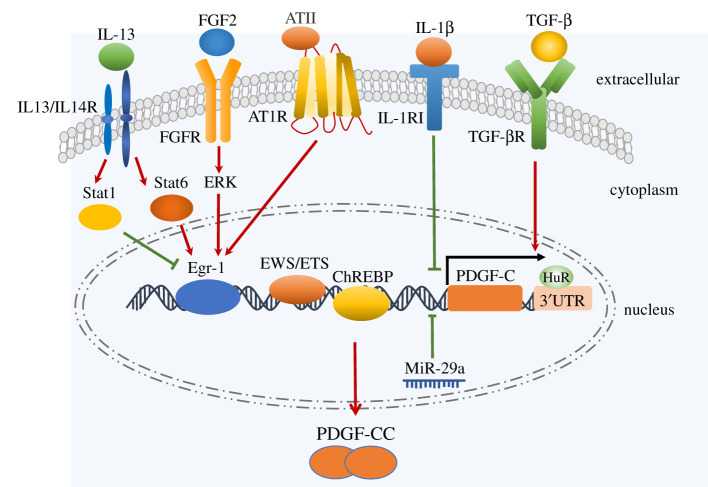
Regulation of PDGF-C expression. Several transcription factors are reported to promote PDGF-C expression, such as EGR1, STAT6, HuR and EWS/FLI. Various factors downregulate PDGF-C expression, such as Sulf2, retinoic acid, MEPMC, IL-1β and microRNA-29a.

Several transcription factors are reported to promote PDGF-C expression, including early growth reactive protein 1 (EGR1), STAT6 [81], HuR (human embryonic lethal abnormal vision-like protein) [107] and EWS/FLI [106] (figure 2). In lung fibroblasts, PDGF-C is upregulated by IL-13 via STAT6 and EGR-1 [81]. In SMCs, PDGF-C is upregulated by EGR-1 through ATII-AT1R or Erk [66,108]. In breast cancer, PDGF-C expression is increased by HuR after its binding to the 3′-untranslated region (3′ UTR) of *PDGF-C* gene [107] (figure 2). In Ewing family tumours, PDGF-C expression is dependent on EWS/FLI fusion protein activity [106]. In human mesangial cells in the kidney, PDGF-C is upregulated by the carbohydrate response element-binding protein (ChREBP) and promotes the development of diabetic nephropathy [109] (figure 2). In addition, it has been shown that PDGF-C can be upregulated by transforming growth factor beta (TGF-β) [110,111] (figure 2).

PDGF-C expression can be downregulated by various factors. It has been shown that sulfatase 2 (Sulf2) downregulates PDGF-C expression in breast cancer [112]. In cultured mouse embryos, retinoic acid administration markedly downregulated PDGF-C and PDGFR-α expression, leading to branchial arch malformation and impaired proliferation of mouse embryonic palatal mesenchymal cells (MEPMC) [113,114]. In human retinal pigment epithelial cells, interleukin 1 beta (IL-1β) downregulates PDGF-C and inhibits RPE proliferation and migration [115]. In human hepatic stellate cells, microRNA-29a downregulates PDGF-C to suppress cell migration and proliferation [77]. Thus, modulating these factors may be of usage to regulate PDGF-C expression levels.

## 4. PDGF-C is critical for the development of multiple organs and tissues

It has been shown that PDGF-C has a vital role in embryonic development. Genetic deletion of *Pdgf-c* leads to embryonic lethality in mice on a 129/S background [116]. PDGF-C deficiency results in multiple defects in various organs and tissues, such as in the vascular and neural systems, lung, palate and kidney [6,14,117] (table 5).

**Table 5.** Phenotypes of Pdgf-c and Pdgfr deficient mice.

phenotype	references
*Pdgf-c^−/−^*	
perinatal lethality, complete cleft of the secondary palate, abnormal branchial arches	[116]
reduced choroidal neovascularization and ischaemia-induced retinal neovascularization, reduced retinal ganglion cell survival after optic nerve crush injury	[30,95]
abnormal cerebral vascularization, asymmetry of the cerebral lateral ventricles, abnormal ventricular lining	[138]
reduction of renal fibrosis and leucocyte infiltration in response to unilateral ureteral obstruction, mitigated glomerular injury and hypertension	[12,118]
lung emphysema, reduction of revascularization in ischaemia limbs of diabetes	[118]
*Pdgfr-α^−/−^*	
perinatal lethality, complete cleft palate, connective tissue deficiency	[119]
neural crest origin defects and incomplete cephalic closure, craniofacial abnormality, abnormal meninges, neuronal over-migration in the cerebral cortex, spina bifida	[120]
abnormal cardiac and cephalic neural crest cell development, reduction of cardiac fibroblasts, cardiovascular defects, heart deformity	[121,122]
reduction of hepatic stellate cell activation and liver fibrosis	[123]
lung emphysema, lung hypoplasia, reduction of SMPCs in the lungs	[49]
abnormal gastrointestinal mucosal lining, skeletal defect	[124]
*Pdgfr-β^−/−^*	
perinatal lethality, reduction of neurons in the superior colliculus and hippocampus, abnormal hippocampal spine, brain oedema, exacerbated cerebral damage after cryogenic injury, BBB integrity breakdown after cerebral ischaemia, demyelination	[125–130]
loss of vSMCs and PCs, dilated heart and aorta, anaemia, thrombocytopaenia, microvessel leakage, microaneurysm formation, haemorrhage	[131]
absence of mesangial cells of glomeruli, dilated capillaries, reduction of mesangial cells	[132]
reduced proliferation and migration of skin fibroblasts, skin wound healing defect, reduced adipose tissue neovascularization and chronic inflammation, defective periosteal bone formation and regeneration	[133–135]
enlarged hepatic injury and infarct volume after ischaemic stroke	[136,137]

### 4.1. Vascular development

PDGF-C is essential for the proper development of the vascular network (table 5). Genetic deletion of *Pdgf-c* in both 129/S and C57BL/6 mice caused vascular defects, such as extracranial vessel haemorrhage [116], and abnormal morphology, density and poor SMC coverage of cerebral blood vessels [138]. Genetic deletion of the major receptor for PDGF-C, *Pdgfr-α*, also results in various vascular defects, such as abnormal yolk sac vasculature and extensive bleeding in various organs [139]. Moreover, *Pdgfr-α* mutation in mice impairs the proper development of aortic and the pulmonary vessels [44] (table 5). These data thus demonstrate an essential role of PDGF-C and its receptor during the development of the vascular system.

### 4.2. Neural system

The neural tube is the primitive central structure of the nervous system during embryonic development, from which the brain and spinal cord develop [140]. PDGF-C is required for the development of the neural tube, notochord and the mesenchyme tissues surrounding them [116,138] (table 5). PDGF-C is critical for the migration and survival of neural crest cells, which are vital for CNS development [56]. *Pdgf-c* genetic deletion in 129/S mice leads to multiple developmental defects in the brain, such as oedema and haemorrhage [116]. Loss of *Pdgf-c* in C57BL/6 mice also results in various defects in the neural system, such as wavy neural tube blisters and ventricular malformations with distorted ependymal linings [138] (table 5). Moreover, *Pdgf-c/Pdgfr-α* double knockout mice also display severe CNS defects, such as irregularly shaped cerebral hemispheres, unusually small cerebella and abnormal interhemispheric fissures [120]. Furthermore, PDGF-C has important functions for the formation of meninges and assembly of the glia limitans basement membrane [120]. In addition, PDGFR-α has been shown to be crucial for oligodendrocyte development and production, and promotes their proliferation and migration [28,39,141,142]. PDGFR-β is expressed in the neural system, and *Pdgfr-β* deficient mice are more vulnerable to brain injury [143]. Thus, plenty of data have shown that PDGF-C and its receptors are of particular importance for the development of the neural system.

### 4.3. Lung

PDGF-C plays a critical role in lung development (table 5). PDGF-C overexpression resulted in various defects in the lung and embryonic lethality, including the excessive proliferation of mesenchymal cells, mesenchymal–epithelial disruption and enlarged and immature lungs [144]. Consistently, genetic deletion of *Pdgf-c* in mice caused emphysema [120]. In addition, PDGF-C has been shown to promote proliferation and inhibits apoptosis and differentiation of lung mesenchymal cells [80]. Moreover, PDGF-C prevents the differentiation of distal airway and airspace epithelial cells into type I alveolar epithelial cells, which constitute the structure of the alveoli and mediate gas exchange [145]. Of the two receptors for PDGF-C, PDGFR-β seems to be more important for lung development since PDGFR-β activity is critically required for embryonic lung growth [57], and inhibition of PDGFR-β signalling with antisense oligodeoxynucleotides significantly reduced embryonic lung epithelial growth and lung size [57,146]. In addition, PDGFR-α signalling has been shown to be vital for lung alveolarization [147]. Thus, both PDGF-C and PDGFRs are critical for lung development.

### 4.4. Palate and kidney

The mouse palate forms at E11.5 from the maxillary processes and mainly comprises epithelial and mesenchymal cells [113,116]. Genetic deletion of *Pdgf-c* or blocking PDGF-C with neutralization antibody leads to palate branchial arch abnormalities, complete cleft palate and embryonic lethality [113,116] (table 5). Consistently, genetic deletion of the major receptor for PDGF-C, *Pdgfr-α*, also results in cleft palate [116]. By contrast, loss of *Pdgfr-β* does not cause cleft palate, suggesting a unique role of the PDGF-C–PDGFR-α axis in palate development. In addition, PDGF-C plays important roles in kidney development by promoting the formation of ureteric buds and mesangial cells in the glomerulus as well as the maturation of kidney arteries and arterioles [8,64]. PDGFR-α is highly expressed in kidney interstitial cells and arterial and venous vessels, suggesting a role of PDGFR-α in kidney development [148]. Genetic deletion of *Pdgfr-β* leads to glomerular mesangial cell failure in mice, demonstrating critical roles of PDGFR-β in glomerular morphogenesis [148].

## 5. PDGF-C and its receptors are expressed in various types of stem cells

Stem cells generally include embryonic stem cells (ESCs), adult stem cells (ASCs) and induced pluripotent stem cells (iPSCs). ESCs are isolated from the ICM of E3.5 embryos [149]. These pluripotent stem cells form derivatives of all the three germ layers except the trophectoderm [149]. ASCs are multipotent stem cells found in adult tissues and can differentiate into various cell types [150]. iPSCs are adult somatic cells reprogrammed by overexpressing the key transcription factors octamer-binding transcription factor 4 (OCT4), sex-determining region Y-box 2 (SOX2), MYC proto-oncogene (c-MYC) and Kruppel-like factor 4 (KLF4) with differentiation capacities similar to ESCs [151–153]. PDGF-C, PDGFR-α and PDGFR-β are expressed in both mouse and human ESCs [23,25,154–156]. PDGF-C expression is also found in various ASCs, such as human mesenchymal stem cells (MSCs) [68], adipose-derived stem cells [157] and vascular stem/progenitor cells [158,159]. Both PDGFR-α and PDGFR-β are expressed in human MSCs [160], adipose-derived stem cells [161–163], vascular stem/progenitor cells [69,164] and neural stem cells (NSCs) [165–167]. PDGFR-β is also expressed in human haematopoietic stem cells (HSCs) [168] and mouse spermatogonial stem cells [169]. In mouse iPSCs, PDGF-C is also expressed as revealed by microarray analysis [170]. PDGFR-β, but not PDGFR-α, is detected in both human and mouse iPSCs [171,172]. Thus, the expression of PDGF-C and its receptors in various types of stem cells suggests potential effects of them in stem cell regulation.

## 6. Effects of PDGF-C and PDGFRs on stem cells

### 6.1. Adult stem cell (ASC)

PDGF-C and the PDGFRs have been demonstrated to have important effects on various types of ASCs (table 6). It has been shown that PDGF-C promotes human MSC proliferation and maintains their multipotency by activating PDGFR-α signalling [68] (table 6). Also, in mouse MSCs, PDGF-C promotes MSC migration via PDGFR-α- and PDGFR-β-induced PI3 K signalling [185]. In addition, it is reported that PDGF-C regulates mouse adipose-derived stem cells and subsequently promotes hair growth [157]. Consistently, PDGFR-α has been shown to promote the proliferation of mouse dermal CD24^+^ adipose-derived stem cells and therefore maintains the adipocyte precursor cell population [163]. Moreover, PDGFR-β is reported to promote the proliferation of human adipose-derived stem cells [161]. Furthermore, PDGF-C also plays important roles in the regulation of vascular stem/progenitor cells. PDGF-C overexpression increased endothelial progenitor cell (EPC) proliferation, migration and adhesion [158]. PDGF-C also induced EPCs to differentiate into ECs and SMCs, thereby promoting the revascularization of ischaemic tissues [69,186]. In addition, it has been shown that PDGF-C activates PDGFR-α in human bone marrow-derived AC133^+^CD34^+^ cells and induce their differentiate into ECs and SMCs [95]. Thus, plenty of data have demonstrated a critical role of PDGF-C in ASC regulation.

**Table 6.** Effects of PDGF-C and PDGFRs on stem cells.

	PDGF-C	PDGFR-α	PDGFR-β	references
ESC		differentiation	differentiation	[156,173,174]
		anti-apoptosis	anti-apoptosis	
		pluripotency	pluripotency	
		proliferation	proliferation	
MSC	multipotency	proliferation	proliferation	[68,175,176]
	proliferation	migration	migration	
		differentiation		
ASC	differentiation	proliferation	proliferation	[161,163,177]
		differentiation	migration	
EPC	proliferation	proliferation	proliferation	[178–182]
	migration	differentiation	migration	
	adhesion		survival	
	differentiation			
NSC		differentiation	multipotency	[166,183,184]
			proliferation	
			survival	

### 6.2. Embryonic stem cell (ESC)

PDGF-C is highly expressed in the very early stage of embryonic development [22–24], and genetic deletion of *Pdgf-c* leads to embryonic lethality [116]. Moreover, both receptors for PDGF-C, PDGFR-α and PDGFR-β, are highly expressed in ESCs [23,25,154–156], further suggesting potential effects of PDGF-C on ESCs. It has been shown that PDGFRs are critical for the undifferentiated state of human ESCs, since inhibition of the PDGFRs downregulated the master pluripotency factors NANOG and OCT4 and led to ESC differentiation [173] (table 6), suggesting a potential role of PDGF-C in maintaining ESC pluripotency. Moreover, it is reported that PDGFR-induces ERK activation inhibits ESC apoptosis [187]. On the other hand, other studies reported that the PDGFR signalling induces differentiation of ESCs into various cell types. For example, inhibition of PDGFR-α by microRNA-218 (miR-218) suppressed ESC migration and differentiation [188], while upregulation of PDGFR-α by mix-like protein 1 (Mixl1) induced ESC differentiation into mesendoderm cells [189]. Moreover, it has been shown that PDGFR-α induces ESC differentiation into blood cells [190], and PDGR-β activation by cyclic strain induces ESC differentiation into vascular SMCs [191]. Furthermore, PDGFR-β is reported to activate the STAT5 and phosphatidylinositol-3 kinase (PI3 K) pathways and induce ESC differentiation into bone marrow cells [192]. These observations thus suggest possible effects of PDGF-C on ESCs and warrant further studies to look into it.

## 7. Concluding remarks

Since the discovery of PDGF-C about two decades ago, studies have demonstrated its critical roles in embryonic development. Loss or overexpression of PDGF-C lead to various developmental defects in multiple organs and tissues, such as in the neural system, palate, lung, kidney and the vasculature. In addition, PDGF-C and its receptors are abundantly expressed in various types of stem cells, such as ESCs, ASCs and iPSCs. PDGFRs have been amply demonstrated to regulate stem cell pluripotency or differentiation, thus suggesting a possible role of PDGF-C in these processes. Future studies are warranted to verify whether and how PDGF-C plays a role in stem cell regulation, particularly, in neural, lung, palate or kidney progenitor/stem cells. It is also critical to identify the regulatory factors governing PDGF-C expression, the discovery of which might lead to new possibilities of therapeutic interventions for developmental defects or stem cell therapy.

## Data accessibility

This article has no additional data.
